# Continuum-Spectral Modeling of Surface Roughness in Electron-Beam-Deposited GO/Ag Nanocomposite Thin Films

**DOI:** 10.3390/nano16070419

**Published:** 2026-03-30

**Authors:** Seyedeh Soheila Mousavi, Milad Mousavi, Davood Raoufi, Ágota Drégelyi-Kiss

**Affiliations:** 1 Department of Physics, Faculty of Science, Bu-Ali Sina University, Hamedan 65174, Iran; s.mousavi@sci.basu.ac.ir (S.S.M.); raoufi@basu.ac.ir (D.R.); 2 Bánki Donát Faculty of Mechanical and Safety Engineering, Óbuda University, 1034 Budapest, Hungary; miladmousavi@stud.uni-obuda.hu

**Keywords:** surface roughness, nanocomposite thin films, electron-beam deposition, power spectral density (PSD), hurst exponent, fractal/multifractal analysis, continuum modeling

## Abstract

This study investigates the structural, chemical, and morphological characteristics of electron-beam–deposited GO/Ag nanocomposite thin films and establishes a compact continuum–spectral framework for quantifying their post-deposition roughness. Since atomic force microscope (AFM) measurements provide only the final, frozen morphology and no direct temporal information, distinguishing between transient and stationary spectra is not experimentally feasible within the limited AFM wavenumber band. In practice, the accessible power spectral densities (PSDs) show no resolvable deviation from the stationary form, and transient contributions cannot be uniquely identified. The stationary PSD is fitted directly to azimuthally averaged AFM spectra, allowing the smoothing coefficients, noise intensity, correlation length, and crossover scale to be extracted in a fully data-driven manner. The fitted model accurately reproduces the characteristic dual (k^−2^)/(k^−4^) spectral scaling and predicts the scan-size dependence of root-mean-square roughness, typically achieving logarithmic determination coefficients above 0.98. The close agreement among parameters obtained from spatially separated sampling points confirms the lateral uniformity of the deposited films and highlights the robustness of the continuum–spectral approach for data-guided roughness control in electron-beam-grown nanocomposite coatings.

## 1. Introduction

Electron-beam-deposited GO/Ag nanocomposite thin films are increasingly explored in both industrial and biomedical technologies [1], where their combined electrical, mechanical, and antimicrobial properties enable multifunctional surface performance [2]. These coatings show strong potential for use in plasmonic and optoelectronic components [3], wear-resistant protective layers, and bio-interfaces such as antimicrobial surfaces, sensing platforms, and implant-adjacent protective coatings [4]. Across such industrial and biomedical implementations, precise control of nanoscale surface morphology becomes critical for tuning interfacial phenomena and ensuring reliable, long-term device performance. Post-deposition surface roughness in functional nanocomposite coatings is not merely a cosmetic artifact; it is a critical design variable governing multiple performance metrics. The morphology of the outermost surface directly influences optical scattering and absorption [5], charge transport pathways [6], interfacial adhesion and delamination resistance [7], friction and wear behavior [8], corrosion initiation [9], and even wettability [10] at the device or contact interface.

Across a wide range of nanostructured materials, such as amorphous silicon thin films used in photovoltaics and metal/oxide nanocomposites, optimal performance is achieved only when a specific, reproducible surface state is stabilized after growth [11], rather than being minimized or left uncontrolled during deposition [12]. For instance, in thin silicon-based absorbers, conversion efficiency improves when the deposition process is tuned to produce a controlled smoothing of the surface, since nanoscale roughness modulates both optical coupling and carrier recombination at the interface.

The same reasoning applies to engineered protective and functional coatings. The final, frozen-in topography encodes the distribution of reinforcing and matrix phases at the surface, which in turn dictates frictional response, local current-carrying pathways, and initiation sites for mechanical or electrochemical failure [13]. However, the surface morphology of electron beam (EB)-deposited and plasma-grown nanocomposite films is intrinsically multiscale and self-affine, producing hierarchical textures in which roughness characteristics vary with observation scale [14].

In such systems, the use of a single root-mean-square (RMS) roughness value, σ, is insufficient, because σ depends strongly on scan length and therefore does not represent an intrinsic physical property. As a result, a scale-aware set of descriptors—such as the 2D power spectral density S(k), correlation length ξ, the Hurst exponent H, fractal dimension Df = 3 − H, and multifractal spectrum f(α)—is required for accurate characterization.

Continuum models provide a powerful connection between the physics of thin-film growth and observable roughness. The seminal linear model of Ortiz et al. [15] describes kinetic roughening via curvature-driven relaxation (∇^2^h) and surface-diffusion smoothing (∇^4^h), predicting spectral features such as k^−2^/k^−4^ scaling and a characteristic crossover length. Subsequent developments extended these ideas to more complex growth equations with variable exponents, nonlinear diffusion, and temperature-dependent relaxation [16].

Experimentally, it has been shown that deposition parameters—energy flux, substrate temperature, and nucleation kinetics—significantly modify surface evolution in materials such as Zr [17], Ni [18], and TiO_2_ [19] thin films. Meanwhile, power spectral density (PSD)-based fractal descriptors have emerged as essential tools for describing self-affine roughness in metallic, semiconducting, and hybrid coatings [20].

Despite these advances, a major gap persists. Most existing studies treat continuum models and PSD/fractal descriptors separately. Continuum models are often used only for qualitative interpretation or for forward simulation of dynamic growth, while PSD/fractal metrics are used only as post-growth descriptors. There is currently no unified framework that (i) fits continuum parameters (a, b, D) directly to experimental atomic force measurement (AFM) PSDs, (ii) predicts scale-dependent RMS roughness σ(L) in a physically consistent way, and (iii) integrates fractal and multifractal descriptors within the same formulation for EB-evaporated nanocomposite films.

Although PSD-based roughness analysis and continuum growth models have been used in prior thin-film studies, these approaches are typically applied separately or only loosely connected [21]. In contrast, the present work develops a unified, data-driven continuum–spectral framework that quantitatively links the fitted relaxation parameters (a, b, ξ) to the observed dual-slope PSD behavior and the independently derived fractal descriptors (H, Df). This approach extracts effective smoothing coefficients directly from stationary PSD fits without requiring transient simulations, predicts the scan-size dependence of the RMS roughness σ(L) with high fidelity, and enables mutual validation between continuum-predicted scaling regimes and fractal metrics in GO/Ag nanocomposite films. This integrated continuum–spectral–fractal methodology provides a more physically grounded and reproducible tool for interpreting and controlling nanoscale roughness in hybrid coatings.

Recent developments in graphene oxide (GO)-based systems have underscored the critical role of nanoscale surface morphology in governing tribological performance, wear resistance, and interfacial durability. For example, the incorporation of GO nanoparticles as eco-friendly additives in wind turbine gearbox lubricants has been shown to significantly suppress wear, reduce friction, and promote surface restoration through optimized flow dynamics and self-healing mechanisms at contact interfaces [22]. These advancements highlight the need for quantitative, scale-aware descriptors of roughness that can link underlying physical mechanisms to measurable surface behavior.

In this work, we address this gap by developing a unified continuum–spectral framework that quantitatively links physically meaningful smoothing parameters to experimentally measured AFM spectra of EB-deposited GO/Ag films. A linear stochastic growth–relaxation model is solved in Fourier space and fitted directly to PSDs to extract (a, b, D), the correlation length ξ = √(b⁄a), and the full scan-size-dependent RMS roughness σ(L). This approach avoids the need for full transient growth simulations and enables direct post-growth prediction and control of surface morphology. Fractal parameters (H, Df) and the multifractal spectrum f(α) further validate the consistency of the continuum description. Lateral uniformity is confirmed by modest variations in (a, b, D, ξ) across spatially separated sampling points.

This study introduces an integrated continuum–spectral–fractal analysis framework for EB-deposited GO/Ag nanocomposite films. While continuum growth models, PSD-based roughness descriptors, and fractal metrics have each been used separately in the literature, they are combined here in a unified, data-driven post-growth characterization approach tailored specifically to EB-evaporated nanocomposite surfaces.

## 2. Materials and Methods

### 2.1. Linear Continuum Model

The post-deposition surface is described by a linear stochastic continuum equation that combines curvature-driven relaxation $\left(a\nabla^{2}h\right)$ with diffusion-driven smoothing $\left(-b\nabla^{4}h\right)$ [15,23]:

(1) ∂h∂t=a∇2h−b∇4h+η(r,t)

where a and b denote the effective surface tension and surface diffusion coefficients, respectively, and η(r,t) is Gaussian white noise with variance D, where r denotes the in-plane spatial coordinate and t denotes time. The corresponding mode relaxation rate depends on the wavenumber.

(2) $$\omega \left(k\right)=bk^{4}+ak^{2}$$

where k is the wavenumber.

### 2.2. Fourier-Space Solution and Spectral Form

For a flat initial condition h(t=0)=0, the mode amplitude obeys

(3) $$\partial_{t}{\hat{h}}_{k}=-\omega \left(k\right){\hat{h}}_{k}+{\hat{\eta }}_{k}<{\hat{\eta }}_{k}(t){\hat{\eta }}_{\overset{´}{k}}(\overset{´}{t})>=2D\delta_{k,-\overset{´}{k}}\delta (t-\overset{´}{t})$$

The mode amplitude ${\hat{h}}_{k}$ is the Fourier transform of the surface height at wavenumber k, which is the amplitude of the fluctuations in Fourier space. The mode relaxation rate ω(k) is the rate at which these modes decay in time due to surface diffusion and curvature-driven processes. ${\hat{\eta }}_{k}$ is the Gaussian white noise in Fourier space, which is the random fluctuations that occur during deposition, with variance D proportional to the deposition flux.

The PSD at deposition time T is given by the following:

(4) $$S(k,T)=\frac{D}{\omega \left(K\right)}[1-e^{-2\omega \left(K\right)T}]\;S_{\infty }(k)=\frac{D}{ak^{2}+bk^{4}}(T\to \infty )$$

For small values of k, the spectral density evolves gradually towards the steady-state value $S_{\infty }(K)$. In contrast, for large values of k, the spectral density S(k, T) quickly decreases $S_{\infty }\left(K\right)$ due to the rapid exponential decay.

The power spectral density (PSD), denoted as S(k), is obtained from the Fourier transform of the height–height correlation function [24].

A spectral crossover occurs at $K_{*}=\sqrt{a/b}$, i.e., correlation length $\xi =\sqrt{b/a}$, for $k\ll k_{*},S\sim k^{-2}$ and for $k\gg k_{*},S\sim k^{-4}$ [15].

**Figure 1:**
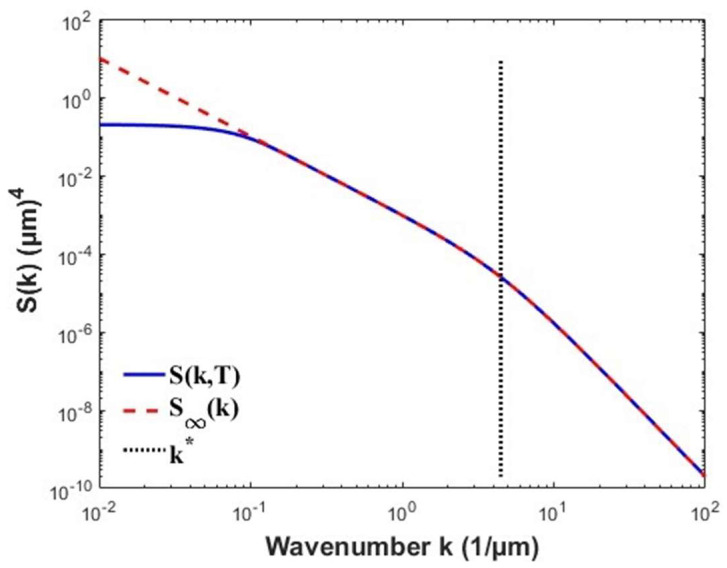
Theoretical PSD at finite time and in the stationary limit.

Theoretical stationary and finite-time spectra are predicted in Figure 1 for a number of k values, demonstrating the fast convergence of the high-k modes to their long-time limit.

### 2.3. Band-Limited RMS Roughness

The experimentally measurable (band-limited) RMS roughness depends on both the deposition time *T* and the AFM scan window. It is defined over the AFM-accessible band [kmin(L), kmax]

(5) $$\sigma^{2}(L,T)=\frac{1}{2\pi }\int_{k_{min}}^{k_{max}}\mathrm{kdk}S(k,T)$$

Here, $k_{min}(L)=\frac{2\pi }{L}$ is set by the scan size L, while kmax is determined by the lateral sampling resolution ∆ (and, if relevant, tip effects), typically $k_{max}\approx min(\frac{\pi }{∆},\frac{c}{r_{tip}}$).

**Figure 2:**
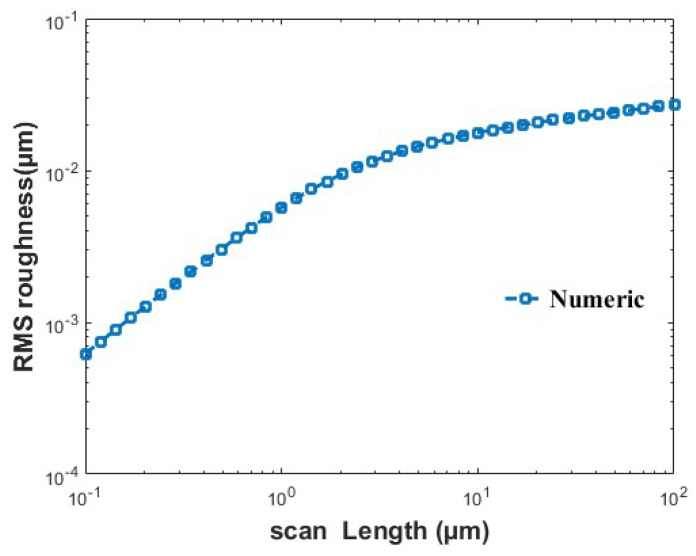
Band-limited RMS roughness versus scan length from numerical evaluation of PSD.

In the stationary regime (T→∞), the same integral is evaluated using $S_{\infty }(k)$. Accordingly, we denote the stationary-limit roughness as σ(L)≡σ(L,∞). This formulation makes the RMS explicitly dependent on the AFM scan size and resolution, and it is computed numerically to maintain full consistency with experimental conditions. The scan size dependence of the band-limited RMS roughness, using the stationary spectrum *S*_∞_(*k*), is shown in Figure 2. This calculation demonstrates that σ(L) increases with scan length and saturates when *k_min_*(L) drops below the lowest resolved band.

### 2.4. Scaling Descriptors from PSD and AFM

Self-affine surfaces exhibit a PSD scaling of the form

(6) $$S_{exp}\left(k\right)\propto k^{-(2H+2)}$$

Here, Equation (6) represents a local self-affine power-law scaling applied over a wavenumber interval where the measured PSD is approximately linear on a log–log plot. Because the continuum model exhibits a crossover at $k^{*}$, the PSD is not characterized by a single exponent over the full band; therefore, H is estimated within the selected quasi-power-law region(s) by fitting logS(k) versus log(k) so that the Hurst exponent is obtained from the slope m of the azimuthally averaged log–log PSD as $H=\frac{-m-2}{2}$, $D_{f}=3-H$, where $D_{f}$ is the fractal dimension of the surface, beyond single-exponent scaling, heterogeneity across scales is quantified by the multifractal spectrum f(α), extracted from AFM height maps using box-counting or wavelet-leader algorithms. Broader spectra (larger Δα) indicate stronger local variability in scaling, often linked to compositional clustering in nanocomposite films [13]. Small departures from the ideal −2 and −4 slopes may arise from finite sampling or mild surface heterogeneity.

### 2.5. Materials

Graphene oxide (GO) and the GO/Ag nanocomposite were synthesized using concentrated sulfuric acid ($H_{2}SO_{4}$), potassium permanganate $(KMNO_{4}$), 30% hydrogen peroxide solution (H_2_O_2_, 30%), hydrochloric acid (HCL), high-purity graphite powder, and pure silver metal (Ag, 99.99% purity) as the starting reagents, all of which were procured from Aldrich Chemical Company (St. Louis, MO, USA).

### 2.6. Methods

Certain analysis techniques were utilized to study the size, shape, and structural characteristics of the synthesized nanocomposite. The XRD pattern was measured on a Unisantis XMD300 diffractometer (Singapore). Field emission scanning electron microscopy images were recorded using the TESCAN MIRA3 XMU system (Brno, Czech Republic). Elemental mapping and energy-dispersive X-ray spectroscopy (EDX) (Abingdon, UK) were used to recognize elemental composition following the same procedure. An electron-beam physical vapor deposition (Ebeam-PVD) machine (Crawley, UK), i.e., vacuum coating unit model 15F6, was used to deposit the nanocomposite thin films. FESEM cross-sectional imaging using a TESCAN MIRA3 XMU system was used to quantify the film.

The GO/Ag nanocomposite thin films were deposited by electron-beam physical vapor deposition (EB-PVD) using a vacuum coating unit (model 15F6). Polished Si(100) wafers with a native oxide layer served as substrates and were ultrasonically cleaned in acetone, ethanol, and deionized water, then dried with nitrogen prior to loading. Deposition was performed at room temperature under a base pressure of 2 × 10^−5^ Torr (working pressure ~5 × 10^−5^ Torr). The pre-synthesized GO/Ag nanocomposite powder was used as the evaporation target. The electron-beam current and acceleration voltage were maintained at 60 mA and 3.8 kV, respectively. The deposition rate was held at 0.1 Å/s, monitored in real time using a quartz crystal microbalance. Deposition duration was adjusted between approximately 80–120 min to achieve final film thicknesses of 80–120 nm, as confirmed by cross-sectional FESEM imaging. No intentional substrate heating or bias voltage was applied. The source–substrate geometry produced a conical angular distribution of the arriving flux relative to the substrate normal.

### 2.7. Graphene Oxide Synthesis by Hummers’ Method

Graphene oxide (GO) was synthesized from commercial graphite using Hummers’ method. Briefly, 10 g of graphite powder was added to 230 mL of concentrated H_2_SO_4_ at 20 °C. Then, 30 g of KMnO_4_ was slowly introduced under magnetic stirring, and the mixture was heated to 40 °C and stirred for 1 h. Subsequently, 500 mL of deionized water was added, and the temperature was raised to 100 °C. Afterward, 2.5 mL of 30 wt% H_2_O_2_ and 500 mL of deionized water were added sequentially. The product was washed with a 1:10 HCl solution to remove metal ions, filtered, and repeatedly rinsed with deionized water. Finally, the GO was dispersed by ultrasonication for 2 h, filtered, and dried at 100 °C for 24 h.

### 2.8. Synthesis of GO/Ag Nanocomposites

**Figure 3:**
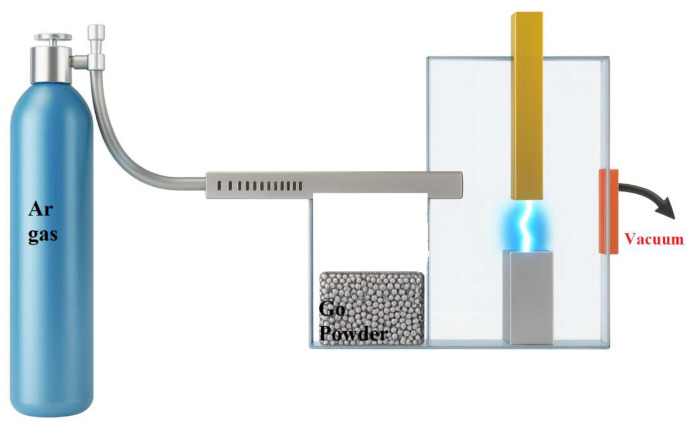
Schematic illustration of the arc discharge setup used for the synthesis of the GO/Ag.

The GO/Ag nanocomposite was synthesized using the electrical arc discharge method under argon gas (99.996% purity) at atmospheric pressure. Argon with a low flow rate was passed through a container holding graphene oxide powder, carrying the GO particles into the discharge chamber. Two silver rods were used as electrodes, and the electrode gap was adjusted until an electrical discharge occurred, leading to the vaporization of silver and the subsequent formation of Ag nanoparticles. The resulting product was collected in powder form on a surface filter using a vacuum pump. A schematic representation of the experimental setup is shown in Figure 3.

### 2.9. AFM Characterization

Surface topographies were measured using a Veeco AutoProbe AFM system (Plainview, NY, USA) operating in tapping mode under ambient conditions. Scans were acquired at three scan sizes (1 × 1 µm^2^, 5 × 5 µm^2^, and 10 × 10 µm^2^) with a pixel resolution of 512 × 512, corresponding to a lateral pixel spacing Δ of approximately 2–20 nm depending on the scan size. At each sampling position (S1–S3), at least three independent scans were collected. Each height map was detrended by subtracting a best-fit plane to remove tilt and then apodized with a 2D Hann window to minimize edge effects prior to Fourier analysis. The two-dimensional power spectral density was computed via fast Fourier transform and physically normalized. The resulting 2D PSD was azimuthally averaged to obtain the isotropic one-dimensional PSD, S(k). For each sampling position, the final PSD used for model fitting was obtained by averaging the azimuthally averaged PSDs from the multiple scans to improve the signal-to-noise ratio. Only the wavenumber band permitted by the scan size and pixel resolution (k_min = 2π/L, k_max ≈ π/Δ) was retained for quantitative analysis and model fitting. The experimentally measured PSD corresponds to the stationary spectrum S_∞_(k) of the continuum model. For the scan sizes used here, π/Δ is always smaller than c/r_tip, so the upper cutoff is set by sampling rather than tip limitations. No tip-induced flattening was observed in the high-k region of the PSD.

### 2.10. Parameter Estimation and Model Fitting

The experimental azimuthally averaged PSD, denoted $S_{exp}\left(k\right)$, was fitted using the stationary form of the linear growth–relaxation model. In the steady regime, the spectrum reduces to:

(7) $$S_{\infty }(k)=\frac{D}{ak^{2}+bk^{4}}$$

with a >0 ,  b>0 ,  and D>0 represent the curvature-driven relaxation coefficient, the surface-diffusion coefficient, and the noise intensity, respectively. This stationary form (7) was used as the fitting model for all samples. Because the AFM window probes only high-k modes whose relaxation times are far shorter than the deposition duration, the measured spectra lie in the saturated regime. Therefore, only the stationary PSD is experimentally identifiable within the accessible band, and transient contributions cannot be resolved. The parameters (a,b,D) were obtained by minimizing the logarithmic least-squares cost function.

(8) $$C=\sum_{i}{\left[{log}_{10}\left(S_{\infty }(k_{i};a,b,D)\right)-{log}_{10}\left(S_{exp}(k_{i})\right)\right]}^{2}$$

Their ratios, rather than their absolute values, carry the physical meaning by setting the correlation length $\xi =\sqrt{b/a}$ and the crossover wavenumber $k^{*}=\sqrt{a/b}$, which separates the $k^{-2}$ and $k^{-4}$ scaling regimes of the stationary PSD. The fitted coefficients *a*, *b*, and D are effective, scale-dependent parameters, and are not intrinsic material constants. Model accuracy was evaluated using the logarithmic coefficient of determination, together with visual inspection of log–log residuals.

(9) $$R_{log}^{2}=1-\frac{\sum_{i}{\left[{log}_{10}S_{exp}(k_{i})-{log}_{10}S_{fit}(k_{i})\right]}^{2}}{\sum_{i}{\left[{log}_{10}S_{exp}(k_{i})-{<log}_{10}S_{exp}>\right]}^{2}}$$

Here, $S_{fit}(k_{i})$ denotes the PSD values predicted by the fitted stationary model, evaluated at the experimental wavenumbers $k_{i}$.

## 3. Results

### 3.1. Structural Validation (XRD, FTIR)

#### 3.1.1. XRD Characterization

**Figure 4:**
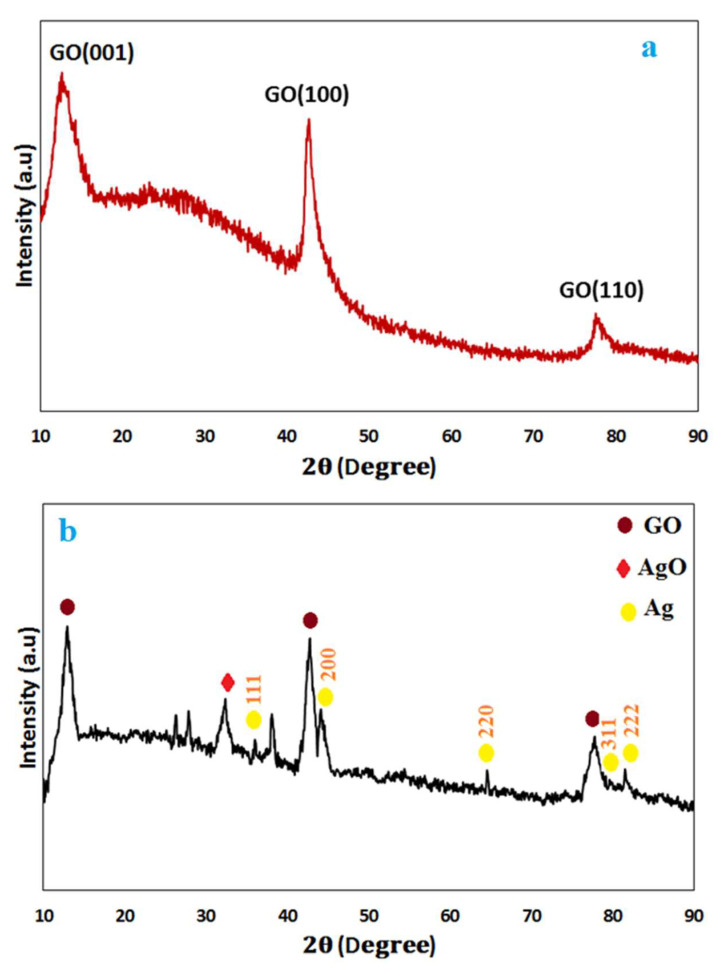
XRD patterns of (**a**) graphene oxide (GO) and (**b**) GO/Ag nanocomposite.

The XRD pattern of graphene oxide (Figure 4a) shows the characteristic peak at (001) around 10–13° (2θ), confirming the enlarged interlayer spacing associated with oxygen-containing functional groups. The in-plane (100) peak appearing at 42–44° indicates residual ordering within the carbon lattice, although its intensity is significantly lower compared to pristine graphite. A weak (110) reflection at (70–80°) further suggests partial in-plane crystallinity, which is disrupted by extensive oxidation [25]. The XRD pattern of the GO/Ag nanocomposite (Figure 4b) confirms the coexistence of both graphene oxide and silver phases. The characteristic GO peak at ~12.5° appears with reduced intensity, attributed to the presence of Ag nanoparticles and disruption of the layered structure. Additional reflections at 46.65° and 77.55° correspond to GO crystallinity. Sharp peaks at 38.2°, 44.4°, 64.42°, 77.4°, and 81.5° indicate the FCC structure of Ag nanoparticles, while a minor peak at 30° suggests the presence of Ag_2_O due to surface oxidation. These results verify the successful integration of both phases in the nanocomposite.

The crystallite size of graphene oxide was estimated using the Scherrer equation,

(10) $$L_{C}=\frac{0.9\;\lambda }{\beta Cos\theta }$$

(11) $$\beta =\frac{FWHM\times 3.1416}{180}$$

where *L_C_* denotes the average crystallite size in order to avoid confusion with the noise intensity D used in the continuum model, λ = 0.154 nm, β is the peak broadening (in radians), and θ is the Bragg angle.

**Table 1:** Data obtained from the Scherrer equation for characteristic peaks in the XRD pattern of graphene oxide.

Samples	Peaks	2θ	FWHM	Spacing	d−D(nm)
1	001	12.6869°	0.3936	6.97758	20.085
2	100	42.62°	0.2952	2.12124	28.56
3	110	78.6852°	0.6888	1.25914	14.75

The broadened GO peaks and the nanoscale Ag inclusions seen in the XRD patterns indicate a granular microstructure.

#### 3.1.2. FTIR Spectroscopy

**Figure 5:**
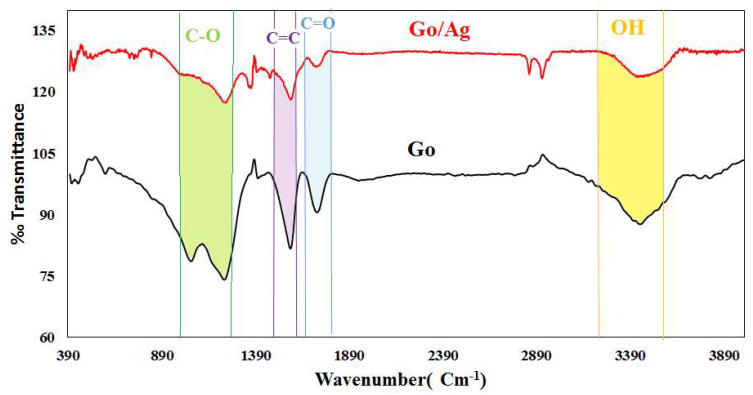
Fourier-transform infrared (FTIR) spectrum of graphene oxide.

The FTIR spectra of GO and the GO/Ag nanocomposite are presented in Figure 5. The spectrum of pristine GO exhibits the characteristic vibrational bands of oxygen-containing functional groups, including the broad O–H (~3200–3500 cm^−1^), C=O (~1720 cm^−1^), C=C (~1575–1610 cm^−1^), and C–O (1050–1220 cm^−1^). These bands confirm the presence of hydroxyl, carbonyl, epoxy, and carboxyl groups typical of oxidized graphene sheets. In the GO–Ag nanocomposite, reduced O–H intensity and shifted/attenuated C=O bands indicate strong interactions with Ag nanoparticles, while additional signals below 700 cm^−1^ confirm Ag–O/Ag–C bonding. These spectral changes verify the successful formation of the GO–Ag nanocomposite with a modified chemical structure. The interaction between GO functional groups and Ag nanoparticles suggested by the FTIR spectrum supports the formation of surface clusters, which naturally contribute to the multiscale height variations detected by the PSD analysis.

### 3.2. SEM/EDS Analysis

**Figure 6:**
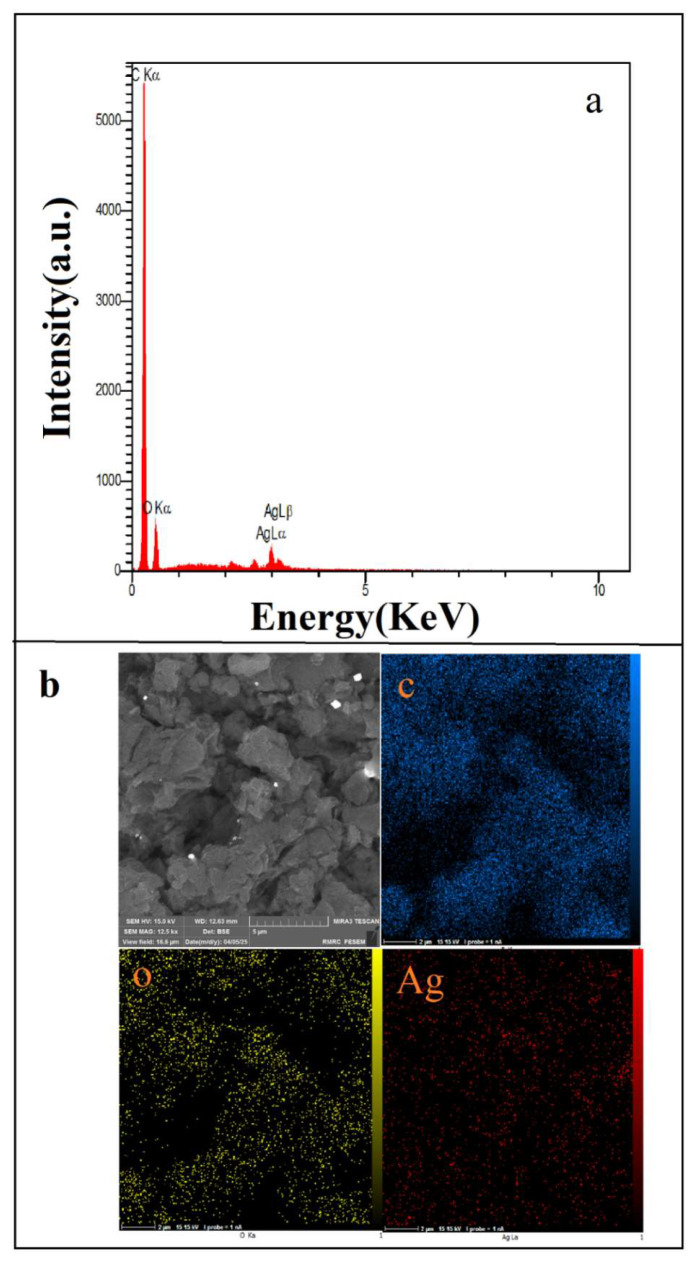
(**a**) EDS spectrum of the GO/Ag nanocomposite (**b**) SEM image and elemental maps showing the spatial distribution of C, O, and Ag.

EDS and SEM analyses of the GO/Ag nanocomposite are presented in Figure 6. The EDS spectrum, shown in Figure 6a, identifies the characteristic Ag Lα and Ag Lβ peaks besides C and O signals due to the graphene oxide matrix, which confirms the successful incorporation of silver within the composite. The SEM micrograph represented in Figure 6b depicts a granular surface morphology, which is in good agreement with the formation of Ag nanoparticles anchored onto the GO sheets. The corresponding elemental mapping images, given in Figure 6, illustrate the spatial distribution of C, O, and Ag throughout the surface.

The granular morphology and the absence of oriented features in the SEM images confirm that the surface lacks directional texture, supporting the use of the isotropic continuum model. The lateral homogeneity of Ag distribution observed in the EDS maps also agrees with the modest spatial variations in the fitted parameters (a, b, D, ξ).

### 3.3. Electron-Beam Deposition and Film Cross-Section GO/Ag Nanocomposites

**Figure 7:**
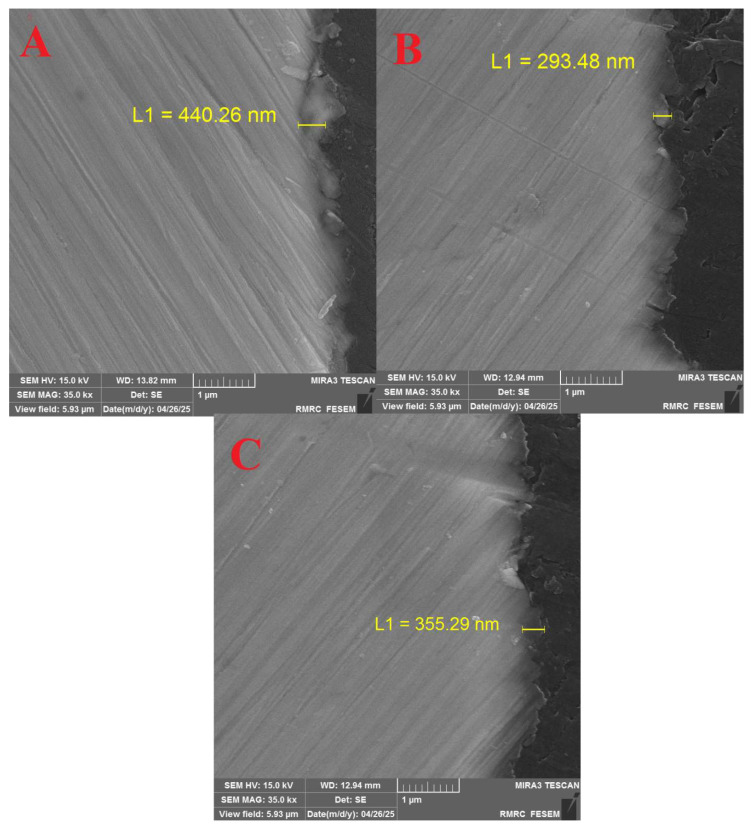
(**A**) Cross-sectional SEM image of sample L1 = 440.26 nm. (**B**) Cross-sectional SEM image of sample L1 = 293.48 nm. (**C**) Cross-sectional SEM image of sample L1 = 355.29 nm.

Electron-beam (EB) evaporation was performed in a high-vacuum chamber with a base pressure of 2 × 10^−5^ Torr and a working pressure of 5 × 10^−5^ Torr. The deposition rate was maintained at 0.1 Å/s and monitored by a quartz crystal microbalance. The electron-beam current and acceleration voltage were 60 mA and 3.8 kV, respectively. The beam was continuously focused on the target to ensure stable evaporation. Due to source–substrate geometry, the arriving flux exhibited a conical angular distribution relative to the substrate normal. Cross-sectional SEM was used to determine film thickness. A schematic of the substrate geometry is shown in Figure 7. The cross-sectional images show that the deposited GO/Ag nanocomposite layer forms a dense and uniform structure with continuous interfaces and good adhesion to the substrate. The columnar morphology is consistent along the thickness, and the absence of large voids indicates stable evaporation and efficient packing of the deposited species. These features denote the effectiveness of EB deposition in producing compact and well-adhered nanocomposite films.

### 3.4. Surface Topography (AFM) and Derived Roughness Parameters

**Figure 8:**
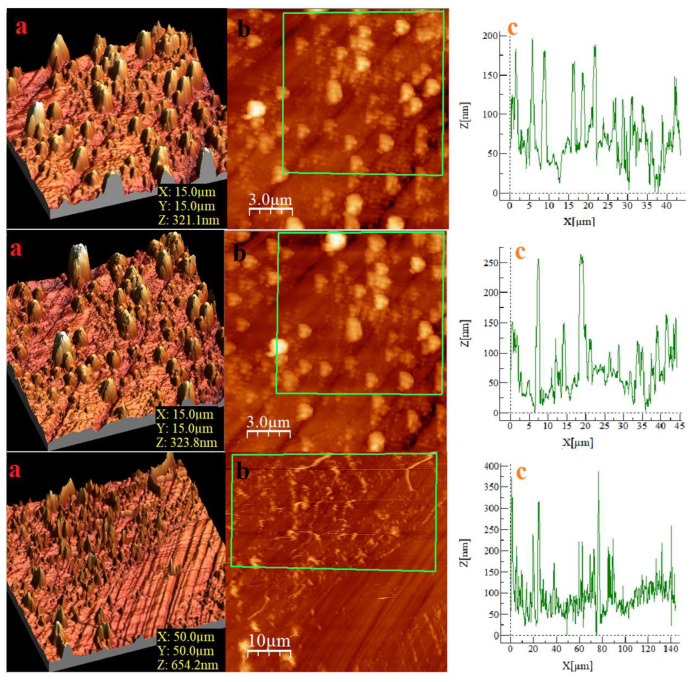
AFM surface topographies of the GO/Ag films at different scan sizes. (I–III) correspond to increasing scan areas. For each scan size: (**a**) 3D height maps, (**b**) 2D amplitude images, and (**c**) representative line profiles.

Representative AFM height maps of the EB-deposited GO/Ag films are shown in Figure 8 for three scan sizes. The images reveal a granular morphology with height variations on both short and intermediate lateral scales, consistent with Ag nanoparticle anchoring on GO sheets. These AFM topographies were used as the raw input for quantitative roughness analysis. Before spectral processing, each height map was detrended by removing a best-fit plane, and no additional filtering or smoothing was applied. The line profiles shown in Figure 8c, extracted along representative horizontal cross-sections at the mid-height of each AFM image, illustrate the variability of peak–valley amplitudes across the scanned region. While these real-space images provide qualitative insight into the morphology, all quantitative metrics—including the PSD, dual-slope scaling, band-limited RMS roughness, and the fitted continuum parameters—were obtained exclusively from the Fourier-domain analysis described. This ensures that the visual interpretation of AFM images is fully consistent with the model-based characterization presented in the subsequent sections.

### 3.5. PSD Scaling, Correlation Length, and Continuum-Model Fitting (Slopes, k*, ξ, Hurst Exponent)

**Figure 9:**
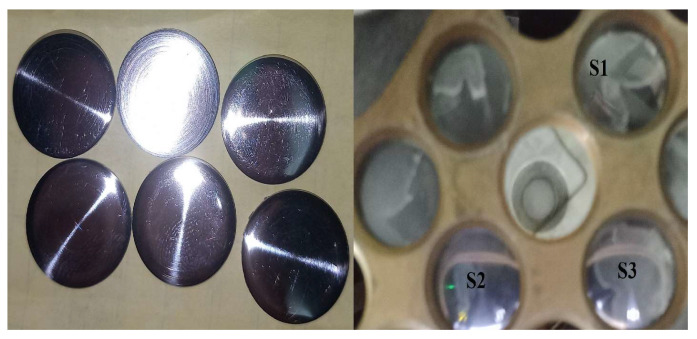
Optical images of the deposited GO/Ag samples (**left**) and the corresponding sampling positions on the substrate holder (**right**), labeled as S1–S3, from which the AFM and PSD analyses were performed.

The azimuthally averaged PSDs extracted from the AFM height maps were fitted to the stationary continuum-model spectrum described in Section 2.6. Across the AFM wavenumber band, the measured spectra do not exhibit the low-k flattening characteristic of the finite-time form S(k,T). Instead, the PSDs already follow the expected $k^{-2}/k^{-4}$ scaling without any detectable transient rollover. This indicates that the experimentally observable bandwidth lies within the stationary regime of the linear model. Figure 9 shows the experimental spectra for the three sampling positions (S1–S3), which correspond to the same substrate locations shown in Figure 8, together with the corresponding best-fit stationary curves. Figure 9 shows the corresponding locations of these sampling points on the substrate holder. All spectra exhibit the characteristic dual $k^{-2}/k^{-4}$ scaling, with a clear crossover separating the curvature-dominated and surface-diffusion-dominated regimes. The fitted smoothing correlation length (ξ), crossover wavenumber (k*), Hurst exponent (H), fractal dimension ($D_{f}$), and the logarithmic determination coefficient $R_{log}^{2}$ for each sample are summarized in Table 2.

**Figure 10:**
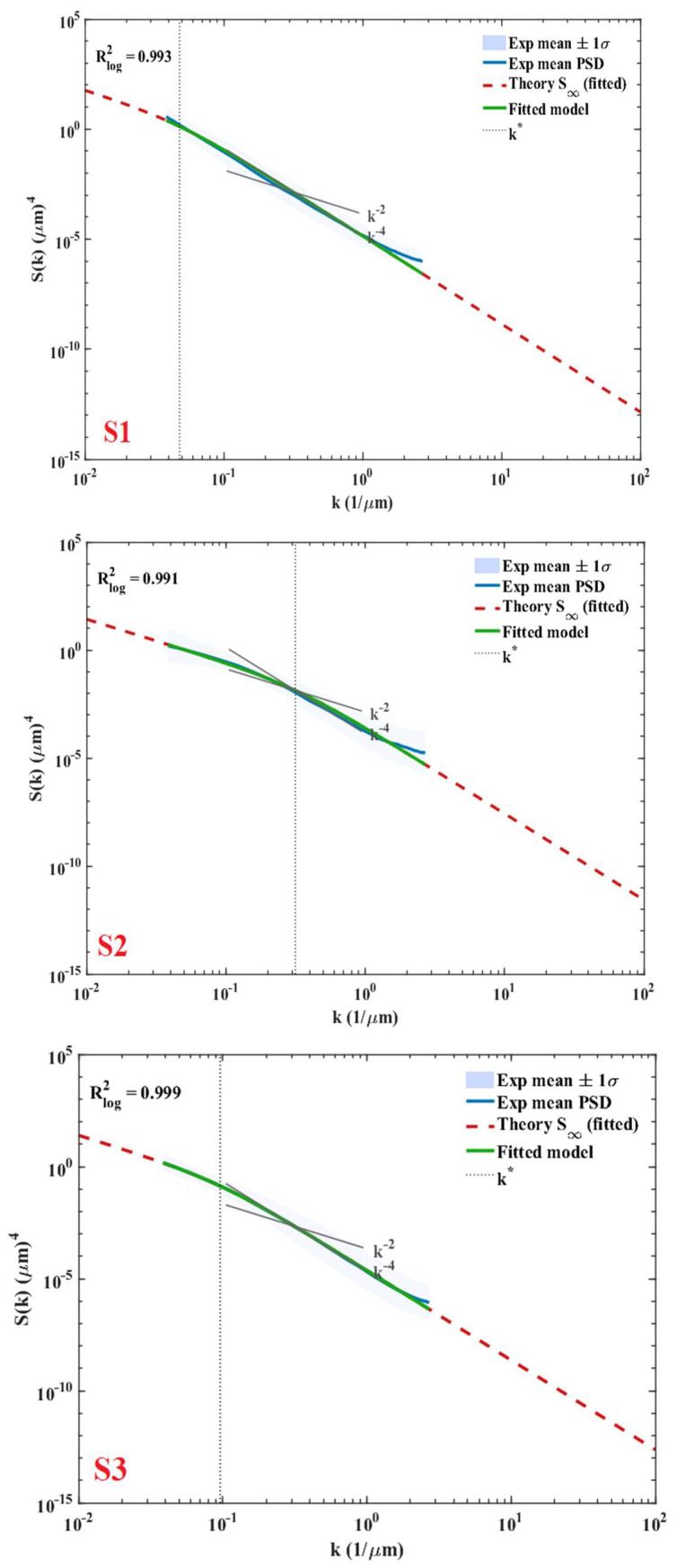
Experimental PSDs of samples S1–S3 with the corresponding fitted stationary model curves.

**Figure 11:**
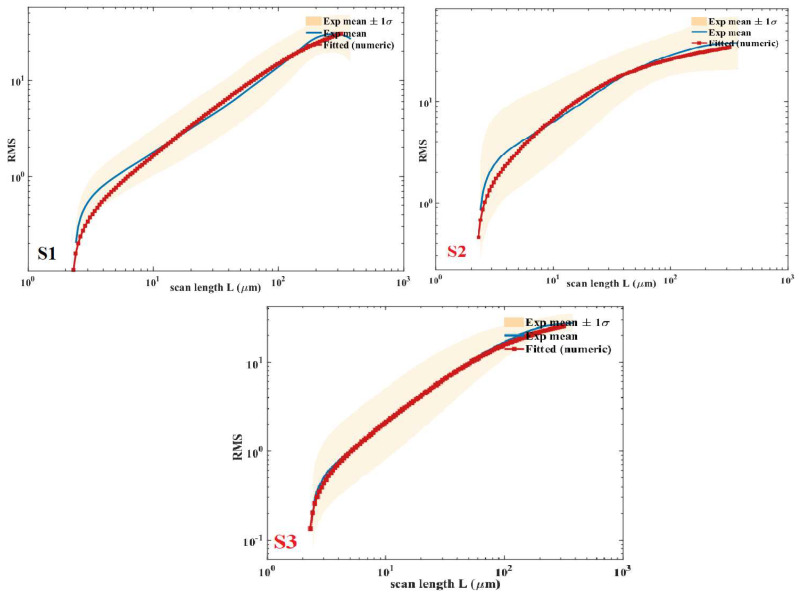
Experimental RMS roughness as a function of scan length L for samples S1–S3, compared with the RMS predicted by the fitted stationary continuum model.

Although the samples originate from different substrate locations, only modest variations are observed in the fitted parameters. These differences are consistent with minor angular variations in the EB evaporation flux. The close agreement in a/b ratios, correlation lengths, and noise intensities indicates that the deposition process maintains a largely uniform roughening–smoothing regime across the examined region. To verify that the fitted parameters reproduce the theoretically expected spectral behavior, the stationary PSD corresponding to the fitted (a, b, D) was computed numerically. Consistent with the reasoning in Section 2.6, the AFM spectra show no measurable deviation from the stationary form within the accessible wavenumber band. The finite-time correction term (1−exp(−2ω(k)T)≈1) is effectively unity for all experimentally accessible modes. The result is shown in Figure 11, confirming the expected dual-slope structure generated by the fitted coefficients.

The accuracy of the fit of the continuum model was further tested by examination of the residuals in the logarithmic domain between the experimental and theoretical PSDs.

## 4. Discussion

The combined structural, chemical, and morphological characterizations provide mutually consistent evidence for successful GO/Ag nanocomposite formation and for its translation into a stable, isotropic post-deposition roughness state.

A deeper examination of the fitted continuum parameters and the independently derived fractal descriptors confirms the mutual consistency across the three analytical layers: continuum modeling, PSD scaling, and fractal analysis. The crossover wavenumber, k* = a/b obtained from the stationary PSD fits delineates the transition from curvature-dominated $k^{-2}$ scaling at low k to diffusion-dominated $k^{-4}$ scaling at high k. The measured Hurst exponents (H ≈ 0.72–0.95 across samples S1–S3) fall within the intermediate range expected for this blended regime, where the AFM-accessible high-k band captures contributions from both relaxation terms rather than the pure asymptotic limits (H ≈ 1 for ∇^2^h alone and H ≈ 0 for ∇^4^h alone). The corresponding fractal dimensions $D_{f}$ = 3 − H (≈ 2.05–2.28) align with the PSD-derived local slopes, further validating the predicted dual-slope behavior. Moreover, the correlation length $\xi =\sqrt{b/a}$ (≈3–21 nm) identifies the characteristic lateral scale of the spectral crossover, and the observed variation in local scaling (reflected in H) independently corroborates this transition. This quantitative agreement demonstrates that the continuum parameters not only reproduce the experimental PSD but also constrain and are themselves validated by the fractal characteristics of the surface, thereby substantiating the unified continuum–spectral–fractal framework introduced in this study.

The fitted parameters a, b, and D in the linear continuum model carry clear physical interpretations in the context of the EB-PVD process. The coefficient a quantifies the strength of curvature-driven relaxation (∇2h), arising from local surface tension and capillary forces that smooth long-wavelength height fluctuations generated during Ag nanoparticle anchoring. The coefficient b represents the magnitude of surface-diffusion-driven smoothing (∇4h) and reflects the effective mobility of adatoms and small clusters on the GO/Ag surface during and immediately after deposition. Under the low deposition rate (0.1 Å/s) and near-room-temperature substrate conditions used here, the extracted b values indicate moderate but finite surface diffusion, consistent with the hybrid organic–inorganic nature of the nanocomposite and the granular morphology observed in SEM. The noise intensity D corresponds to the stochastic deposition flux, incorporating random arrival of GO sheets and Ag nanoparticles, variations in cluster size, and local nucleation events. Together, the ratio b/a determines the correlation length ξ = √(b⁄a) and the crossover wavenumber k* = √(a⁄b), which define the characteristic lateral scale separating curvature-dominated and diffusion-dominated regimes. These parameters therefore provide a direct, physically meaningful bridge between the EB-PVD process conditions and the resulting multiscale surface topography of the GO/Ag films.

To quantitatively verify that the AFM-accessible wavenumber band lies within the stationary regime, we estimated the characteristic transient-rollover wavenumber $k_{trans}$ from the condition 2 λ(k) T ≈ 1, where λ(k) = $bk^{4}+ak^{2}$ is the fitted mode-relaxation rate, and T is the deposition duration. Using the extracted parameters (a,b) from the stationary PSD fits, $k_{trans}$ was found to be at least one order of magnitude smaller than the experimental lower cutoff kmin=2πLscan across all scan sizes (with L extending up to several micrometers). Consequently, for all experimentally accessible modes (k ≥ $k_{min}$), the condition 2 λ(k) T ≫ 1 is satisfied, demonstrating that the absence of observable low-k rollover is not merely a limitation of the measurement bandwidth but reflects the intrinsically rapid relaxation of high-k modes relative to the deposition timescale, thereby rigorously supporting the conclusion that the measured PSDs correspond to the stationary regime.

The applicability of the linear Ortiz-type model (Equation (1)) to the present GO/Ag nanocomposite films is supported by the isotropic granular morphology revealed in SEM and EDS analyses. The surface exhibits no columnar structures, directional textures, or pronounced composition-dependent clustering—features that typically indicate nonlinear growth mechanisms such as slope-dependent deposition, shadowing, or compositionally modulated diffusion. The absence of these signatures suggests that curvature-driven (∇^2^h) and diffusion-driven (∇^4^h) relaxation processes dominate the post-deposition surface evolution, rendering the linear formulation an appropriate first-order description. In this context, the fitted parameters a and b represent effective coefficients that capture the dominant relaxation physics within the AFM-accessible wavenumber band. Had strong nonlinearities been operative, they would likely manifest as deviations from the predicted k − 2/k − 4 dual-slope structure, anomalous Hurst exponents, or poor fit quality—none of which are observed here. This agreement supports the conclusion that the linear model provides a physically consistent and experimentally well-justified representation of the roughness evolution in these EB-deposited GO/Ag films.

The physical meaning of the correlation length ξ = b/a can be further understood by comparing it with the microstructural features observed in SEM. The extracted values of ξ (≈3–21 nm across samples S1–S3) are of the same order as the characteristic lateral spacing between Ag-rich granular clusters visible in the SEM images. This suggests that ξ represents the dominant lateral smoothing scale associated with the interplay between Ag nanoparticle anchoring and the underlying GO-sheet topology. In the continuum framework, ξ marks the length scale at which curvature-driven and diffusion-driven relaxation contribute equally to the surface evolution. The agreement between this theoretically defined crossover scale and the experimentally observed granular spacing provides a physically meaningful interpretation of ξ and reinforces the consistency between the continuum parameters and the film’s microstructure.

**Figure 12:**
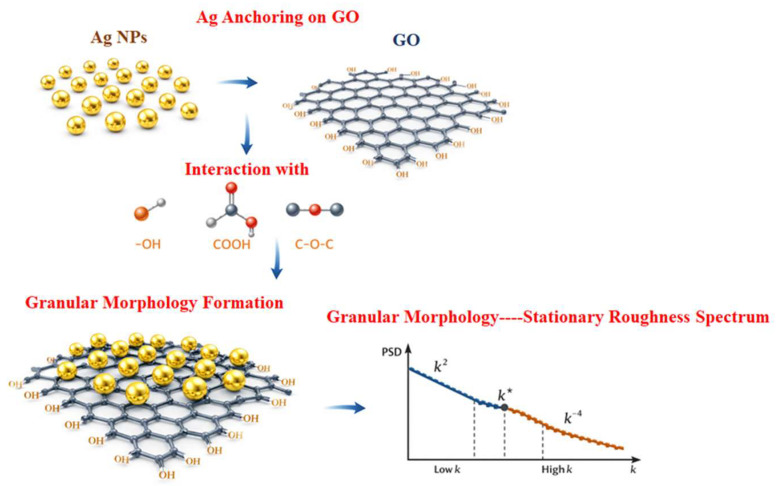
Coherent picture of GO/Ag film formation and roughness stabilization.

XRD confirms that the GO host is preserved while undergoing Ag-induced structural modification, and simultaneously verifies the crystallization of Ag nanoparticles within/on the GO framework [26]. FTIR complements XRD by identifying the chemical bonding environment responsible for Ag anchoring and for the observed changes in the GO lamellar structure. After Ag incorporation, attenuation and/or shifts in the O–H and C=O bands indicate that hydroxyl and carbonyl/carboxyl sites participate in interfacial interactions with Ag nanoparticles. Functional groups that expand and hydrate the interlayer spacing are partially consumed or chemically altered when they coordinate Ag, promoting partial restacking/compaction and changing the effective lamellar order [27]. Morphological evidence from SEM/EDS and AFM provides a consistent spatial picture of how the GO/Ag chemistry and structure manifest as a granular, laterally uniform, and largely isotropic surface. EDS spectra identify Ag together with C and O, and elemental mapping demonstrates that Ag is distributed laterally across the surface without strong clustering at the scale resolved by mapping, supporting uniform incorporation by the synthesis/deposition route. AFM topographies further confirm granular morphology at the nanoscale [28]. The absence of strongly oriented features in both SEM and AFM supports the isotropy assumption used in azimuthal PSD averaging and in adopting an isotropic linear continuum framework for the post-deposition surface. This causal chain—Ag anchoring on GO, granular nanoparticle-mediated roughening, and subsequent stabilization by smoothing mechanisms—is summarized schematically in Figure 12.

The PSD analysis provides the quantitative bridge between morphology and the continuum surface-dynamics model, converting the granular/isotropic visual morphology into physically interpretable, scale-resolved descriptors. The azimuthally averaged AFM PSDs exhibit the characteristic dual scaling behavior, transitioning between k^−2^ and k^−4^ regimes with a clear crossover scale. This is exactly the spectral predicted by the linear stochastic growth–relaxation equation with curvature-driven relaxation (∇^2^h) and surface-diffusion smoothing (∇^4^h), where the crossover wavenumber k* and correlation length ξ separate the long-wavelength curvature-dominated behavior from short-wavelength diffusion-dominated smoothing. Importantly, within the AFM-accessible band, no measurable low-k flattening associated with finite-time transients is resolved; the spectra follow the stationary form without detectable deviation. The high logarithmic coefficients of determination and the modest parameter variation across spatially separated sampling points demonstrate both goodness of fit and lateral uniformity of the deposited films, consistent with the homogeneous Ag distribution observed by EDS mapping. From an engineering perspective, this unified continuum–spectral characterization is valuable because it converts AFM measurements from a scan-size-dependent roughness number into physically meaningful, scale-aware control parameters. The demonstrated reproducibility of fitted parameters across the substrate suggests that EB deposition can produce nanocomposite films with consistent roughness physics over the sampled area, which is essential for device reliability and scalable coating performance. The main limitation of the present analysis is intrinsic to AFM bandwidth: AFM provides only the final morphology and restricts measurable spatial frequencies to a finite k-window set by scan size and sampling resolution. As a result, discriminating transient from stationary spectra is not experimentally feasible if the accessible band does not include the low-k rollover region where finite-time effects would appear. Future work can address these points by extending scan sizes to lower k, using complementary in situ or time-resolved probes (or multi-technique roughness measurements) to better constrain transient evolution, and performing controlled parametric deposition studies to map how processing conditions tune the continuum parameters and the PSD crossover. Such extensions would further strengthen the predictive use of the continuum–spectral framework for targeted roughness control in EB-grown nanocomposite coatings.

## 5. Conclusions

The combined structural, chemical, morphological, and spectral analyses provide a coherent and comprehensive validation of the GO/Ag nanocomposite films produced by electron-beam deposition. XRD and FTIR measurements confirm the successful incorporation of Ag nanoparticles into the graphene oxide matrix, SEM and EDS analyses demonstrate a granular, isotropic surface morphology and a laterally uniform Ag distribution. AFM measurements reveal multiscale height variations that originate from these nanoscale features. A compact, continuum-spectral framework was developed that quantitatively described and fitted the post-deposition roughness of EB-deposited GO/Ag nanocomposite thin films. The fitted PSDs reproduce the dual-slope scaling behavior and predict the scan-size dependence of RMS roughness. The approach provides a reproducible data-driven method for roughness control without full dynamic simulation and establishes a bridge between surface physics and AFM observables. The results demonstrate that electron-beam deposition is a robust and effective method for producing compact, chemically integrated GO/Ag nanocomposite films with well-defined isotropic roughness. The combination of experimental characterization and continuum-spectral modeling offers both mechanistic insight and practical predictive capability, enabling rational control of nanocomposite film morphology for future technological applications.

## Figures and Tables

**Table 2:** Quantitative summary of the continuum–spectral model parameters for different sample positions on the substrate holder.

Value	ξ=ba	k∗=ab	H	$D_{f}$	$R_{log}^{2}$
$s_{1}$	20.7764	0.0481	0.8872	2.1127	0.992
$s_{2}$	3.167	0.3156	0.7246	2.2753	0.991
$s_{3}$	10.4089	0.0960	0.9455	2.0544	0.999

## Data Availability

The data is available through the corresponding author.

## Nomenclature

**h(x,y):**	**Surface height (μm)**
**S(K):**	**Azimuthally averaged PSD vs. spatial wavenumber k (μm^−1^)**
**σ:**	**Band-limited RMS roughness (μm)**
**H:**	**Hurst exponent: *D_f_* = 3 − H (surface)**
**f(α):**	**Multifractal spectrum**
**ξ:**	**Correlation length =** ba **(μm)**
**a, b:**	**Linear smoothing coefficient (unit such that** $\omega \left(k\right)=ak^{2}+bk^{4}$**)**
**D:**	**Noise intensity proportional to deposition flux**
**T:**	**Deposition duration (s)**
***k_min_*, *k_max_*:**	**AFM spectral band limits**

## References

[1] Liu C., Fang C., Shao C., Zheng X., Xu H., Huang Q. (2021). Single-step synthesis of AgNPs@ rGO composite by e-beam from DC-plasma for wound-healing band-aids. Chem. Eng. J. Adv..

[2] Cobos M., De-La-Pinta I., Quindós G., Fernández M.J., Fernández M.D. (2020). Graphene oxide–silver nanoparticle nanohybrids: Synthesis, characterization, and antimicrobial properties. Nanomaterials.

[3] Attia R., Yousif N., Balboul M. (2022). Facile synthesis of r GO/Ag/PVA nanocomposites polymeric films by electron beam irradiation for optoelectronic applications. Opt. Mater..

[4] Wang S., Liu S., Cao S., Bao Y., Wang L., He Z.E., Li J., Zhou Y., Lv M. (2024). Engineering bacterial biofilm development and structure via regulation of silver nanoparticle density in graphene oxide composite coating. JACS Au.

[5] Li F., Klepzig L.F., Keppler N., Behrens P., Bigall N.C., Menzel H., Lauth J. (2022). Layer-by-layer deposition of 2D CdSe/CdS nanoplatelets and polymers for photoluminescent composite materials. Langmuir.

[6] Jiao H., Li Y., Niu X., Ji X., Xia J., Zhang J., Cheng X., Wang Z. (2024). High performance La1-xAlxF3 nanocomposite coatings prepared by a co-evaporation technique. Opt. Express.

[7] Chen H.Y., Chen Z.Y., Mao M., Wu Y.Y., Yang F., Gong L.X., Zhao L., Cao C.F., Song P., Gao J.F. (2023). Self-adhesive polydimethylsiloxane foam materials decorated with MXene/cellulose nanofiber interconnected network for versatile functionalities. Adv. Funct. Mater..

[8] Piotrowska K., Madej M., Kowalczyk J., Radoń-Kobus K. (2023). Surface roughness effects on the properties of silicon-doped Diamond-like Carbon coatings. Coatings.

[9] Zhang Q., Feng Y., Liao W., Li J., Yin C., Zhou J., Chen Z., Zhang P., Ning Z. (2023). Preparation and corrosion resistance of superhydrophobic Ni–Co–Al_2_O_3_ coating on X100 steel. RSC Adv..

[10] Wang M., Xue Z., Yan S., He J., Shao Q., Ge W., Lu B. (2022). Pulse electrodeposited super-hydrophobic Ni-Co/WS_2_ nanocomposite coatings with enhanced corrosion-resistance. Coatings.

[11] Hussain M.Z., Yang Z., Huang Z., Jia Q., Zhu Y., Xia Y. (2021). Recent advances in metal–organic frameworks derived nanocomposites for photocatalytic applications in energy and environment. Adv. Sci..

[12] Coe S.C., Wadge M.D., Felfel R.M., Ahmed I., Walker G.S., Scotchford C.A., Grant D.M. (2020). Production of high silicon-doped hydroxyapatite thin film coatings via magnetron sputtering: Deposition, characterisation, and in vitro biocompatibility. Coatings.

[13] Zhou W., Cao Y., Zhao H., Li Z., Feng P., Feng F. (2022). Fractal analysis on surface topography of thin films: A review. Fractal Fract..

[14] Shakoury R., Matos R.S., da Fonseca Filho H.D., Rezaee S., Arman A., Boochani A., Jurečka S., Zelati A., Mardani M., Ţălu Ş. (2023). Investigation of deposition temperature effect on spatial patterns of MgF2 thin films. Microsc. Res. Tech..

[15] Ortiz M., Repetto E., Si H. (1999). A continuum model of kinetic roughening and coarsening in thin films. J. Mech. Phys. Solids.

[16] Ghosh A., Velázquez J.J. (2024). A thin film model for meniscus evolution. J. Math. Fluid Mech..

[17] Annamuradov B., Khuzhakulov Z., Khenner M., Terzic J., Gurgew D., Er A.O. (2025). Substrate Temperature-Induced Crystalline Phase Evolution and Surface Morphology in Zirconium Thin Films Deposited by Pulsed Laser Ablation. Coatings.

[18] Sun H., Cui J., Wang H., Yang S., Xaikoua S., Tan Y., Zhou X., Wang B., Sun J. (2023). Effect of temperature on electronucleation and growth mechanism of Zn–Ni alloy in deep eutectic solvent. Anti-Corros. Methods Mater..

[19] Galhenage R.P., Yan H., Tenney S.A., Park N., Henkelman G., Albrecht P., Mullins D.R., Chen D.A. (2013). Understanding the nucleation and growth of metals on TiO_2_: Co compared to Au, Ni, and Pt. J. Phys. Chem. C.

[20] Eftekhari L., Raoufi D., Eshraghi M.J., Ghasemi M. (2022). Power spectral density-based fractal analyses of sputtered yttria-stabilized zirconia thin films. Semicond. Sci. Technol..

[21] Bontempi M., Visani A., Benini M., Gambardella A. (2020). Assessing conformal thin film growth under nonstochastic deposition conditions: Application of a phenomenological model of roughness replication to synthetic topographic images. J. Microsc..

[22] Su J., Ouyang Y., Yin L., Shi Z., Zhou C., Wang H., Hu B. (2025). Advancing wind turbine gearbox durability with eco-friendly GO nanolubricants: CFD simulation–experiment synergy for understanding flow dynamics, wear suppression and surface restoration mechanisms. Tribol. Int..

[23] Kryukov Y., Podraza N., Collins R., Amar J. (2009). Experimental and theoretical study of the evolution of surface roughness in amorphous silicon films grown by low-temperature plasma-enhanced chemical vapor deposition. Phys. Rev. B-Condens. Matter Mater. Phys..

[24] Gupta I., Mohanty B.C. (2016). Dynamics of surface evolution in semiconductor thin films grown from a chemical bath. Sci. Rep..

[25] Sajeev V., Rane S., Ghosh D., Acharyya N., Roy Choudhury P., Mukherjee A., Roy Chowdhury D. (2023). Terahertz sensing of reduced graphene oxide nanosheets using sub-wavelength dipole cavities. Sci. Rep..

[26] Kumari S., Sharma P., Yadav S., Kumar J., Vij A., Rawat P., Kumar S., Sinha C., Bhattacharya J., Srivastava C.M. (2020). A novel synthesis of the graphene oxide-silver (GO-Ag) nanocomposite for unique physiochemical applications. ACS Omega.

[27] Faid A.H., Rafea M.A., Gad S., Sharaky M., Ramadan M.A. (2024). Antitumor efficiency and photostability of newly green synthesized silver/graphene oxide nanocomposite on different cancer cell lines. Cancer Nanotechnol..

[28] Hou Q., Wang X., Ragauskas A.J. (2019). Dynamic self-assembly of polyelectrolyte composite nanomaterial film. Polymers.

